# Supplementary material for: Role transformation of fecundity and viability: The leading cause of fitness costs associated with beta-cypermethrin resistance in Musca domestica
Source: PLoS One. 2020 Jan 30;15(1):e0228268. doi: 10.1371/journal.pone.0228268 (PMC6992221; doi:10.1371/journal.pone.0228268)
Supplement: S1 Table — (DOCX) [file pone.0228268.s001.docx]

**Supporting information**

**S1 Table. The life table of the CSS and CRR in ≤ 21 days.**

|  |  | CSS |  |  |  | CRR |  |
| --- | --- | --- | --- | --- | --- | --- | --- |
| Strain *x* | *N_x_*(Production) | *d_x_*(Death) | *q_x_*(Mortality) |  | *N_x_*(Production) | *d_x_*(Death) | *q_x_*(Mortality) |
| Adult (N_0_) | 10 |  |  |  | 10 |  |  |
| ♀: ♂ (N_0_) | 1.00 |  |  |  | 1.00 |  |  |
| Egg | 3912.33±115.97 | 1625.67±49.02 | 58.44±0.71 |  | 883.67±44.21*** | 1240.33±51.60** | 71.22±6.33*** |
| Larva | 2286.67±79.87 | 486.33±34.83 | 21.29±1.46 |  | 627.00±22.55*** | 69.00±4.16*** | 11.04±0.83** |
| Pupa | 1800.33±77.06 | 254.67±13.59 | 14.18±0.82 |  | 558.00±23.64*** | 169.33±5.04** | 30.40±0.80*** |
| Adult (N_1_) | 1545.67±74.04 | 7.67±0.33 | 0.50±0.00 |  | 388.67±19.85*** | 8.00±0.58 | 2.06±0.07 |
| ♀×2 | 1500.00±75.27 | 10.67±0.67 | 1.43±0.08 |  | 286.67±14.89 | 14.00±1.15 | 9.74±0.33*** |
| Normal♀×2 | 1492.67±77.64 |  |  |  | 271.33±13.48 |  |  |
| ♀:♂ (N_1_) | 0.94±0.05 |  |  |  | 0.58±0.01*** |  |  |
| Mean Eggs/♀ | 728.47±23.19 | 325.13±9.80 | 41.65±0.71 |  | 176.73±8.84*** | 248.07±10.32** | 28.78±3.66 |
| *R_o_* | 132.07±7.16 |  |  |  | 26.48±1.58 |  |  |
| *r_m_* | 0.57±0.03 |  |  |  | 0.31±0.03** |  |  |
| Fitness (*W*) | 154.57±7.40 |  |  |  | 38.87±1.99*** |  |  |
| Relative Fitness | 1.00 |  |  |  | 0.25 |  |  |
| *C* (%) | - |  |  |  | 44.84 |  |  |

Note: a life table of the CSS and CRR strains in ≤ 21 days. The figures in the table were represented by the mean the total mean and standard error (±SE). *N_x_* is the total production numbers at age *x*, *d_x_* is the death numbers of the individuals at age *x* and *q_x_* is the mortality ratio of initial dying individuals at age *x*. Statistically significant differences between CSS and CRR: **P*<0.05, ***P*<0.01, ****P*<0.001.
