# Supplementary material for: Role transformation of fecundity and viability: The leading cause of fitness costs associated with beta-cypermethrin resistance in Musca domestica
Source: PLoS One. 2020 Jan 30;15(1):e0228268. doi: 10.1371/journal.pone.0228268 (PMC6992221; doi:10.1371/journal.pone.0228268)
Supplement: S2 Table — (DOCX) [file pone.0228268.s002.docx]

**Supporting information**

**S2 Table. The entire life table of the CSS and CRR in ≤ 30 days.**

|  |  | CSS |  |  |  | CRR |  |
| --- | --- | --- | --- | --- | --- | --- | --- |
| Strain *x* | *N_x_*(Production) | *d_x_*(Death) | *q_x_*(Mortality) |  | *N_x_*(Production) | *d_x_*(Death) | *q_x_*(Mortality) |
| Adult (N_0_) | 10 |  |  |  | 10 |  |  |
| ♀: ♂ (N_0_) | 1.00 |  |  |  | 1.00 |  |  |
| Egg | 4240.33±94.41 | 1802.67±48.09 | 42.52±1.61 |  | 1964.67±63.34*** | 1320.33±45.72** | 67.20±1.07*** |
| Larva | 2437.67±75.51 | 544.67±31.47 | 22.37±2.24 |  | 644.33±22.67*** | 85.00±4.04*** | 13.24±1.58** |
| Pupa | 1893.00±73.91 | 274.67±11.26 | 14.55±1.34 |  | 559.33±24.37*** | 170.00±5.29** | 30.44±1.32*** |
| Adult (N_1_) | 1681.33±73.17 | 11±0.58 | 0.68±0.03 |  | 389.33±20.17 | 8.67±0.88 | 2.36±63.85*** |
| ♀×2 | 1569.33±74.70 | 12.67±0.67 | 0.82±0.07 |  | 288.00±15.28 | 15.33±1.76 | 5.63±0.83*** |
| Normal ♀×2 | 1556.67±74.39 |  |  |  | 271.33±13.48^a^ |  |  |
| ♀:♂ (N_1_) | 0.94±0.00 |  |  |  | 0.60±0.00*** |  |  |
| Mean Eggs/♀ | 848.07±18.88 | 360.53±9.62 | 42.52±1.61 |  | 392.93±12.67*** | 264.07±9.14** | 67.20±1.07*** |
| *R_o_* | 137.43±7.10 |  |  |  | 26.50±1.59 |  |  |
| *r_m_* | 0.57±0.05 |  |  |  | 0.31±0.05** |  |  |
| Fitness (*W*) | 161.83±7.32 |  |  |  | 38.93±2.02 |  |  |
| Relative Fitness | 1.00 |  |  |  | 0.24 |  |  |
| *C* (%) | - |  |  |  | 45.29 |  |  |

Note: a total life table of the CSS and CRR strain in ≤ 30 days. The figures in the table were represented by the mean the total mean and standard error (±SE). *N_x_* is the total production numbers at age *x*, *d_x_* is the death numbers of the individuals at age *x* and *q_x_* is the mortality ratio of initial dying individuals at age *x*. Statistically significant differences between CSS and CRR: * *P*<0.05, ** *P*<0.01, *** *P*<0.001. ^a^ indicates that the *N_x_* value of the female adult does not change at the 21~30 day, so it is the same as the *N_x_* value of ≤ 21 day.
