# Supplementary material for: Role transformation of fecundity and viability: The leading cause of fitness costs associated with beta-cypermethrin resistance in Musca domestica
Source: PLoS One. 2020 Jan 30;15(1):e0228268. doi: 10.1371/journal.pone.0228268 (PMC6992221; doi:10.1371/journal.pone.0228268)
Supplement: S3 Table — (DOCX) [file pone.0228268.s003.docx]

**Supporting information**

**S3 Table. Comparison of the life history traits of CSS between in early lifetime (≤ 21 days) and in later lifetime (≥ 21 days).**

| Strain CSS | *N_x_* | | *d_x_* | | *q_x_* | |
| --- | --- | --- | --- | --- | --- | --- |
|  | t | d*f* | t | d*f* | t | d*f* |
| Egg | 30.30^***^ | 2 | 29.47^***^ | 4 | -1.31 | 2 |
| Larva | 25.12^***^ | 4 | 10.07^***^ | 4 | -8.08^***^ | 4 |
| Pupa | 22.05^**^ | 4 | 16.98^***^ | 4 | -5.22^**^ | 4 |
| Adult (N_2_) | 19.85^**^ | 2 | 9.19^***^ | 4 | 115.85^***^ | 4 |
| ♀×2 | 18.98^**^ | 2 | 13.00^**^ | 2 | -12.30^***^ | 4 |
| Normal“♀×2” | 18.36^**^ | 2 | 29.47^***^ | 4 | -1.31 | 4 |
| ♀:♂ | 0.96 | 2 |  |  |  |  |

Note: *N_x_* is the total production numbers at age *x*, *d_x_* is the death numbers of the individuals at age *x* and *q_x_* is the mortality ratio of initial dying individuals at age *x*. Statistically significant differences: * *P*<0.05, ** *P*<0.01, *** *P*<0.001.
