# Supplementary material for: Role transformation of fecundity and viability: The leading cause of fitness costs associated with beta-cypermethrin resistance in Musca domestica
Source: PLoS One. 2020 Jan 30;15(1):e0228268. doi: 10.1371/journal.pone.0228268 (PMC6992221; doi:10.1371/journal.pone.0228268)
Supplement: S4 Table — (DOCX) [file pone.0228268.s004.docx]

**Supporting information**

**S4 Table. Comparison of the life history traits of CRR between in early lifetime (≤ 21 days) and in later lifetime (≥ 21 days).**

| Strain CRR | *N_x_* | | *d_x_* | | *q_x_* | |
| --- | --- | --- | --- | --- | --- | --- |
|  | t | d*f* | t | d*f* | t | d*f* |
| Egg | 17.48^***^ | 4 | 22.26^**^ | 2 | -15.38^***^ | 4 |
| Larva | 27.02^***^ | 2 | 12.61^***^ | 4 | -17.49^***^ | 4 |
| Pupa | 23.53^**^ | 2 | 33.37^***^ | 4 | -0.48 | 4 |
| Adult (N_2_) | 19.53^**^ | 2 | 8.32^***^ | 4 | -0.94 | 2 |
| ♀×2 | 19.08^***^ | 4 | 7.18^**^ | 4 | -0.85 | 2 |
| Normal“♀×2” | 20.13^***^ | 4 | 22.26^**^ | 2 | -14.31^**^ | 2 |
| ♀:♂ | 55.72^***^ | 4 |  |  |  |  |

Note: *N_x_* is the total production numbers at age *x*, *d_x_* is the death numbers of the individuals at age *x* and *q_x_* is the mortality ratio of initial dying individuals at age *x*. Statistically significant differences: **P*<0.05, ***P*<0.01, ****P*<0.001.
