# Supplementary material for: Role transformation of fecundity and viability: The leading cause of fitness costs associated with beta-cypermethrin resistance in Musca domestica
Source: PLoS One. 2020 Jan 30;15(1):e0228268. doi: 10.1371/journal.pone.0228268 (PMC6992221; doi:10.1371/journal.pone.0228268)
Supplement: S5 Table — (DOCX) [file pone.0228268.s005.docx]

**Supporting information**

**S5 Table. Pearson correlation analysis of the CSS.**

|  | Age | Clutches | Fecundity | Fitness | Longevity  ♀ | Longevity  ♂ | Size  first | Size | Viability  first |
| --- | --- | --- | --- | --- | --- | --- | --- | --- | --- |
| Clutches | 0.846* |  |  |  |  |  |  |  |  |
| Fecundity | 0.179 | 0.238 |  |  |  |  |  |  |  |
| Fitness | 0.255 | 0.393 | 0.963** |  |  |  |  |  |  |
| Longevity  ♀ | 0.076 | 0.215 | -0.552 | -0.719 |  |  |  |  |  |
| Longevity  ♂ | 0.493 | 0.326 | -0.734 | -0.618 | 0.384 |  |  |  |  |
| Size first | 0.204 | 0.601 | 0.470 | 0.670 | -0.857* | -0.218 |  |  |  |
| Size | -0.584 | -0.670 | 0.562 | 0.401 | -0.238 | 0.839* | -0.152 |  |  |
| Viability  first | 0.696 | 0.582 | -0.190 | -0.142 | 0.396 | 0.614 | -0.180 | -0.641 |  |
| Viability | 0.301 | 0.495 | 0.884* | 0.977** | -0.813* | -0.495 | 0.792 | 0.254 | -0.097 |

Note: Statistically significant correlation: **P*<0.05, ***P*<0.01.
