# Supplementary material for: Role transformation of fecundity and viability: The leading cause of fitness costs associated with beta-cypermethrin resistance in Musca domestica
Source: PLoS One. 2020 Jan 30;15(1):e0228268. doi: 10.1371/journal.pone.0228268 (PMC6992221; doi:10.1371/journal.pone.0228268)
Supplement: S6 Table — (DOCX) [file pone.0228268.s006.docx]

**Supporting information**

**S6 Table. Pearson correlation analysis of the CRR.**

|  | Age | Clutches | Fecundity | Fitness | Longevity  ♀ | Longevity  ♂ | Size  first | Size | Viability  first |
| --- | --- | --- | --- | --- | --- | --- | --- | --- | --- |
| Clutches | -0.305 |  |  |  |  |  |  |  |  |
| Fecundity | 0.217 | 0.334 |  |  |  |  |  |  |  |
| Fitness | 0.446 | 0.334 | 0.959** |  |  |  |  |  |  |
| Longevity  ♀ | -0.082 | -0.443 | 0.437 | 0.291 |  |  |  |  |  |
| Longevity  ♂ | 0.596 | -0.146 | -0.014 | 0.182 | 0.132 |  |  |  |  |
| Size first | -0.025 | 0.957** | 0.398 | 0.467 | -0.529 | -0.023 |  |  |  |
| Size | 0.426 | -0.361 | 0.759 | 0.718 | 0.738 | 0.088 | -0.268 |  |  |
| Viability  first | 0.480 | 0.247 | 0.707 | 0.742 | 0.162 | 0.195 | 0.377 | 0.529 |  |
| Viability | 0.664 | 0.300 | 0.813* | 0.945** | 0.093 | 0.389 | 0.499 | 0.598 | 0.706 |

Note: Statistically significant correlation: **P*<0.05, ***P*<0.01.
