# Supplementary material for: Role transformation of fecundity and viability: The leading cause of fitness costs associated with beta-cypermethrin resistance in Musca domestica
Source: PLoS One. 2020 Jan 30;15(1):e0228268. doi: 10.1371/journal.pone.0228268 (PMC6992221; doi:10.1371/journal.pone.0228268)
Supplement: S7 Table — (DOCX) [file pone.0228268.s007.docx]

**Supporting information**

**S7 Table. The variation analysis of the fitness and its components between the CSS and CRR.**

|  | CSS | CRR | *t* | d*f* |
| --- | --- | --- | --- | --- |
| Age | 8.33±0.33 | 28.17±0.58 | -4.00^**^ | 10 |
| Clutches | 50.67±0.92 | 11.00±0.60 | 20.49^***^ | 10 |
| Fecundity | 4179.50±69.09 | 1937.67±59.07 | 24.66^***^ | 10 |
| Fitness | 154.52±5.62 | 38.45±2.09 | 19.35^***^ | 6 |
| Longevity♀ | 23.17±0.69 | 20.20±0.38 | 3.78^**^ | 10 |
| Longevity♂ | 24.70±0.31 | 20.23±0.30 | 10.40^***^ | 10 |
| Size first | 131.57±5.38 | 108.57±2.59 | 3.84^**^ | 10 |
| Size | 82.60±1.77 | 68.88±2.19 | 4.87^***^ | 10 |
| Viability first | 32.78±0.61 | 19.60±0.55 | 16.07^***^ | 10 |
| Viability | 36.91±0.78 | 19.78±0.52 | 18.27^***^ | 10 |

Note: Statistically significant differences: **P*<0.05, ***P*<0.01, ****P*<0.001.
