# Supplementary material for: Role transformation of fecundity and viability: The leading cause of fitness costs associated with beta-cypermethrin resistance in Musca domestica
Source: PLoS One. 2020 Jan 30;15(1):e0228268. doi: 10.1371/journal.pone.0228268 (PMC6992221; doi:10.1371/journal.pone.0228268)
Supplement: S8 Table — (DOCX) [file pone.0228268.s008.docx]

**Supporting information**

**S8 Table. The Independent Sample Test results of the mating frequency of CSS and CRR at three fixed times.**

| Day-old after  eclosion (day) | Time-1 | | Time-2 | | Time-3 | |
| --- | --- | --- | --- | --- | --- | --- |
|  | t | d*f* | t | d*f* | t | d*f* |
| Day-2 | -2.74 | 4 | -6.69^**^ | 4 | -2.21 | 4 |
| Day-3 | 0.86 | 4 | -0.62 | 4 | 1.58 | 4 |
| Day-4 | 10.25^***^ | 4 | 0.98 | 4 | 2.40 | 4 |
| Day-5 | 2.16 | 4 | 6.64^**^ | 4 | 0.97 | 4 |
| Day-6 | -0.89 | 4 | -5.63^**^ | 4 | -0.10 | 4 |
| Day-7 | -1.44 | 4 | -4.82^**^ | 4 | -0.61 | 4 |
| Day-8 | -3.18^*^ | 4 | -4.58^**^ | 4 | -3.38 | 2 |

Note: Time-1: the first fixed observing time (9:00 AM); Time-2: the second fixed observing time (3:00 PM); Time-3: the third fixed observing time (9:00 PM). Statistically significant differences between CSS and CRR: **P*<0.05, ***P*<0.01, ****P*<0.001.
