# Supplementary material for: Role transformation of fecundity and viability: The leading cause of fitness costs associated with beta-cypermethrin resistance in Musca domestica
Source: PLoS One. 2020 Jan 30;15(1):e0228268. doi: 10.1371/journal.pone.0228268 (PMC6992221; doi:10.1371/journal.pone.0228268)
Supplement: S9 Table — (DOCX) [file pone.0228268.s009.docx]

**Supporting information**

**S9 Table. The differences of the mating frequency at the three fixed observing times.**

| Day-old after  eclosion (day) |  | CSS |  |  |  | CRR |  |
| --- | --- | --- | --- | --- | --- | --- | --- |
|  | Time-1 | Time-2 | Time-3 |  | Time-1 | Time-2 | Time-3 |
| Day-2 | 1.28 a | 2.56 b | 1.39 a |  | 3.56 a | 4.56 b | 3.39 a |
| Day-3 | 3.28 a | 5.11 b | 3.78 a |  | 2.72 a | 5.44 b | 2.44 a |
| Day-4 | 5.61 a | 7.00 b | 5.94 a |  | 2.39 a | 6.50 c | 3.50 b |
| Day-5 | 4.33 a | 6.44 b | 4.61 a |  | 2.83 a | 3.61 b | 3.94 b |
| Day-6 | 1.56 a | 2.94 b | 1.94 a |  | 1.94 a | 6.17 c | 2.61 b |
| Day-7 | 1.28 a | 1.94 b | 1.17 a |  | 2.00 b | 4.50 c | 1.33 a |
| Day-8 | 1.28 ab | 1.61 b | 0.94 a |  | 2.28 a | 3.89 b | 2.89 a |

Note: Time-1: the first fixed observing time (9:00 AM); Time-2: the second fixed observing time (3:00 PM); Time-3: the third fixed observing time (9:00 PM). Different letters of the same strain indicate significant differences at *P* = 0.05.
